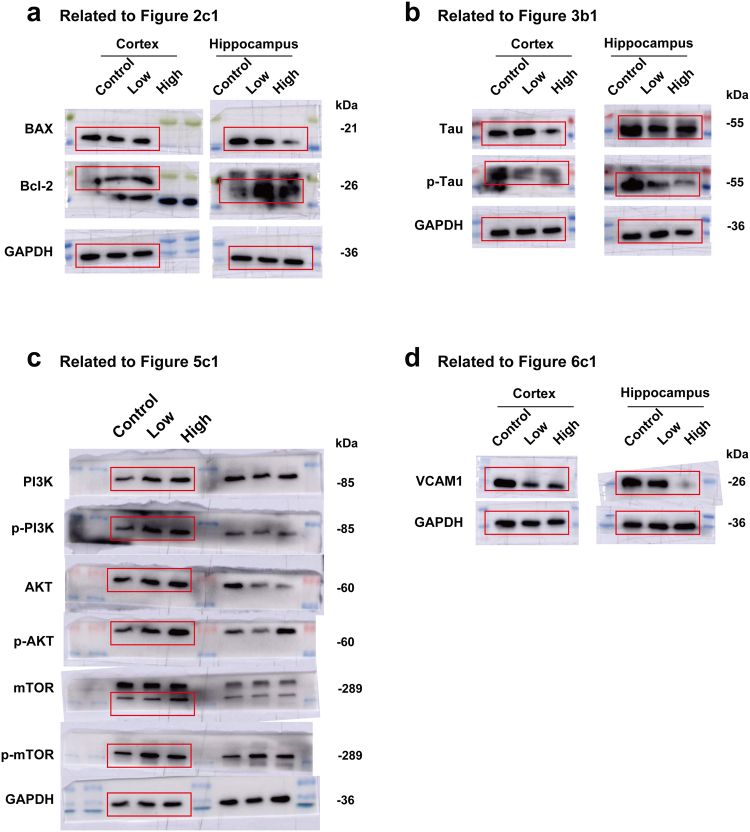# Long-term Omega-3 polyunsaturated fatty acid supplementation improves meningeal lymphatic function during brain aging in mice

**DOI:** 10.1016/j.jlr.2025.100895

**Published:** 2025-09-09

**Authors:** Zhoujing Liu, Jiamin Peng, Xuemin Wang, Fei Yin, Fengjuan Su, Zhong Pei, Hongfu Wu, Chuanming Luo

**Affiliations:** 1 Department of Neurology, The Seventh Affiliated Hospital, Sun Yat-Sen University, Shenzhen, China; 2 Department of Thoracic Surgery, The Seventh Affiliated Hospital, Sun Yat-Sen University, Shenzhen, China; 3 Department of Neurology, The First Affiliated Hospital, Sun Yat-sen University, Guangdong Provincial Key Laboratory of Diagnosis and Treatment of Major Neurological Diseases, National Key Clinical Department and Key Discipline of Neurology, Guangzhou, China; 4 Dongguan Key Laboratory of Stem Cell and Regenerative Tissue Engineering, The First Dongguan Affiliated Hospital, Guangdong Medical University, Dongguan, China

**Keywords:** Omega-3 polyunsaturated fatty acids, meningeal lymphatic system, anti-brain aging

## Abstract

Emerging evidence implicates that meningeal lymphatic dysfunction may contribute to the pathogenesis of brain age-related diseases, suggesting its potential as a therapeutic target for brain aging. This study investigated whether long-term Omega-3 polyunsaturated fatty acids (Omega-3 PUFAs) supplementation could delay brain aging through meningeal lymphatic modulation. We randomly assigned C57BL/6J mice into control, low-dose, and high-dose Omega-3 PUFAs groups, and administered dietary supplementation for 12 months until reaching 24 months of age. We then assessed the anti-aging effects on brain function and further examined meningeal lymphatic performance in clearance capacity and immune regulation. Our findings demonstrate that long-term Omega-3 PUFAs supplementation increases docosahexaenoic acid (DHA) and eicosapentaenoic acid (EPA) levels in the brain, reduces age-related neuronal loss, and improves motor and cognitive behaviors in aged mice. Additionally, it reduces the accumulation of toxic proteins (phosphorylated tau and amyloid-β) and metabolites (NADPH, succinyl-CoA, and cAMP) in the brain and decreases immune cell infiltration (CD68+ microglia and CD3+ T cells) in the central nervous system of aged mice. Furthermore, we demonstrate that these protective effects may be mediated through preservation of the meningeal lymphatic system during aging. In conclusion, this study elucidates a novel understanding of the anti-brain-aging mechanisms of Omega-3 PUFAs.

Improvements in public health care have inadvertently increased the prevalence of geriatric diseases, particularly Alzheimer's disease (AD), driving urgent demand for therapeutic interventions targeting these brain age-related diseases (1). Neurodegenerative pathologies, including neuronal loss and misfolded protein accumulation, are considered to be closely associated with the development of brain age-related diseases (2, 3). These findings underscore the importance of enhancing the clearance of toxic proteins or metabolites in the brain, with recent research highlighting the therapeutic potential of the meningeal lymphatic system—a drainage network discovered in 2015 (4, 5).

The meningeal lymphatic system comprises a network of dural vessels that facilitate the drainage of cerebrospinal fluid (CSF) and molecular solutes into the deep cervical lymph nodes (dCLNs), playing a crucial role in maintaining brain homeostasis (6, 7). Accumulating evidence has demonstrated that this system undergoes significant structural and functional decline with age, such as impaired CSF clearance, potentially exacerbating neurodegenerative processes and influencing the process of brain age-related diseases (8, 9). Notably, enhancing beta amyloid protein (Aβ) clearance through the meningeal lymphatic system has been shown to alleviate AD-like symptoms, positioning this system as a potential therapeutic target (10, 11).

Additionally, widespread chronic neuroinflammation are closely associated with brain age-related diseases (12). The meningeal lymphatic system, draining abundant immune cells, functions as a regional immune center for neuro-immune interactions (13). Comparative analyses reveal age-associated meningeal immune dysregulation, characterized by increased infiltration of immune cells and amplified inflammatory responses that may contribute to neurodegenerative processes (13, 14).

Omega-3 polyunsaturated fatty acids (Omega-3 PUFAs) are the primary components of fish oil, mainly including docosahexaenoic acid (DHA), eicosapentaenoic acid (EPA), and alpha-linolenic acid (ALA) (15). They are indispensable in maintaining structural integrity and function of the central nervous system (CNS) (16, 17). In our previous study on young (8–10 week old) fat-1 transgenic mice (capable of synthesizing Omega-3 PUFAs), it was found that Omega-3 PUFAs could enhance the clearance of fluorescent tracers and exogenous soluble Aβ in CSF (18). However, the mechanistic relationship between long-term Omega-3 PUFAs supplementation, meningeal lymphatic function preservation, and brain aging attenuation remains poorly defined.

This study interrogates how Omega-3 PUFA supplementation augments meningeal lymphatic function in aging, using cardinal features of AD, such as Aβ pathology as a paradigm to dissect nutrient-mediated clearance mechanisms, thereby informing targeted clinical strategies for brain-aging modulation.

## Materials and Methods

### Animals and dietary formulation

Age-matched C57BL/6J mice (12-month-old, 1:1 male: female ratio) were randomized into control, low-dose, and high-dose Omega-3 PUFAs groups and fed for 12 consecutive months. Sample size was dynamically adapted to assay requirements: initial cohorts for non-terminal behavioral tests, subsets for destructive assays. All groups received nutritionally balanced chow with standardized composition meeting AIN-93G specifications (19, 20) (Jiangsu Xietong Pharmaceutical Bio-Engineering Co,Ltd; supplemental Table S1), wherein the experimental diets differed solely in fish oil supplementation (containing 70% DHA and 8% EPA; Wuhan Shengtianyu Biotech Ltd).

(1) Control: AIN-93G diet with no fish oil
(2) Low-dose: AIN-93G diet + 271.43 mg/kg fish oil
(3) High-dose: AIN-93G diet + 1,357.15 mg/kg fish oil

Dietary formulation followed a three-step optimization protocol.

#### Step 1. Target dose determination

Based on previous research, mice given oral administration of 20 mg/kg/d DHA was the low dose (21), and the corresponding concentration of EPA is 2.28 mg/kg/d. The amount of fish oil added in the high-dose was fivefold greater (100 mg/kg/d DHA).

#### Step 2. Feed incorporation

The DHA concentration in feed (mg/kg) was calculated as:

$$\text{DHA}\;\text{concentration}\;\text{in}\;\text{feed}\;\left(\text{mg}/\text{kg}\right)=\frac{\text{Daily}\;\text{DHA}\;\text{intake}\;\left(\text{mg}/\text{kg}\right)\;x\;\text{Average}\;\text{body}\;\text{weight}\;\left(g\right)}{\;\text{Daily}\;\text{food}\;\text{intake}\;\left(g/d\right)}$$

According to the mice's average body weight (30.15 g) and food intake (3.86 g) in our pre-experiment, the DHA content in feed was 156.25 mg/kg in the low dose and 781.25 mg/kg in the high dose.

#### Step 3. Stability adjustment

Considering the potential nutrient degradation during feed processing/storage (22), we implemented a 21% overage adjustment, resulting in final DHA concentrations of 190 mg/kg in low-dose and 950 mg/kg in high-dose. The final fish oil additive levels (271.43 mg/kg and 1,357.15 mg/kg for low- and high-dose groups, respectively) were derived from the 70% DHA content in the fish oil. This rigorous formulation process ensured precise delivery of target Omega-3 PUFA doses throughout the 12-month intervention period.

The study procedures were approved by the ethics committees of the Sun Yat-Sen University (SYSU-IACUC-2022-000342, SYSU-IACUC-2021-000248).

### Fatty acid and metabolic products analysis

Fatty acid quantification was performed via Gas Chromatography-Mass Spectrometry (GC-MS) using methyl salicylate (500 ppm) as an internal standard. Calibration curves (1–2,000 μg/ml) were prepared by serial dilution of stock solutions (4,000 μg/ml) in n-hexane. Cerebral homogenates underwent methanol-sulfuric acid (1% v/v) mediated esterification (80°C, 30 min), followed by n-hexane extraction, ice-water washing, and dehydration with anhydrous sodium sulfate. Analysis employed a TRACE 1310 GC system with TG-FAME column (50 m × 0.25 mm ID × 0.20 μm) and helium carrier (0.63 ml/min) (Shanghai Bioprofile Technology Co., Ltd.). Temperature programming spanned 80°C (1 min) to 250°C (3 min) via incremental ramping. ISQ 7000 mass spectrometric detection operated in SIM mode (70 eV EI) targeting characteristic fragment ions. Quantitation utilized peak area ratios against calibration curves. DHA content was then normalized to the weight of the original brain tissue (μg per g tissue) by multiplying the calculated concentration (μg/ml) by the total extraction volume (ml) and dividing by the tissue weight.

### Intrastriate injection of soluble Aβ

Fluorescein isothiocyanate-conjugated Aβ1-42 oligomers (FITC-Aβ1–42; GL Biochem, Ltd., China) were dissolved to a concentration of 100 mM. Anesthetized animals were fixed in a stereotaxic frame, and a microsyringe (Hamilton Co., Reno) was inserted into the left striatum (AP, 0.22; ML, 22.5; and DV, 3.5 mm). One microliter of FITC-Aβ1–42 was infused slowly, with 5-min post-injection stabilization before needle retraction.

### Morris water maze

Swimming trials were video-recorded using an automated video tracking system (Xinruan Information Technology Co., Ltd) to quantify spatial learning parameters. We analyzed swimming velocity, escape latency (time to locate submerged platform), and escape distance of each mouse. On the day after hidden platform training, the platform was removed and a probe test was performed. Spatial memory was assessed through: (a) platform-crossing frequency, and (b) target quadrant dwell time percentage.

### Open field test

Locomotor activity and anxiety-like behavior were evaluated in a 40 × 40 cm automated arena. Briefly, mice were individually placed in the central zone for 5-min recordings. Key metrics included: (a) average velocity (mm/s), (b) total distance traveled (mm), and (c) center zone entries (defined as >50% body mass in zone).

### Novel object recognition (NOR)

The mice were shown two objects and allowed to explore freely. One of the two objects was replaced with a novel object. The recognition index (time spent exploring the novel object/total time) was chosen as the standard measurement.

### Rota Rod

Motor coordination was evaluated using an accelerated rotational protocol (0–40 rpm over 300 s). Performance was quantified as mean latency to fall across three trials (10-min inter-trial intervals).

### Pole test

Neuromotor function was assessed using a vertical pole apparatus (50 cm height, 1 cm diameter). Briefly, mice were positioned head-upward at the apex, with timed measurements of: (a) time to achieve downward head orientation (T-turn), and (b) time to reach the base platform (T-D). Final scores represented the mean of two trials (15-min recovery period between trials).

### Sample preparation

For molecular analyses, mice were transcardially perfused with 50 ml ice-cold saline. The hippocampus and frontal-parietal cortex were immediately transferred into a lysis buffer (containing protease/phosphatase inhibitor cocktail), homogenized by ultrasonication, and then centrifuged (12,000 rpm for 30 min at 4°C).

For histopathological examination, sequential perfusion was performed with 50 ml ice-cold saline followed by 4% paraformaldehyde (PFA). The brain was immersed in 4% PFA overnight and transferred to 20% and 30% sucrose solutions. Tissue blocks were embedded in OCT compound (Sakura Finetek, #4583) and sectioned coronally at 10 μm thickness using a cryostat (Leica CM 1850 UV; Leica). Brain sections were collected at intervals of 15 pieces and stored at −80°C.

### Western blot assay

30 μg of total protein were separated using 10% SDS-PAGE and subsequently electrotransferred to 0.22 μm PVDF membranes. Membranes were blocked with 5% bovine serum albumin (BSA; #CCS30014.01D, MRC) for 1 h at room temperature, then incubated with corresponding primary antibodies at 4°C overnight. After incubation with horseradish peroxidase (HRP)-conjugated secondary antibodies, the protein was visualized with ECL detection reagent (#WBKLS0500, Millipore), and the densitometry of the bands was analyzed using Image J software.

### Enzyme-linked immunosorbent assay (ELISA)

Tissue homogenates from the frontal-parietal cortex and hippocampus were prepared in ice-cold PBS (1:10 w/v) using probe sonication. After centrifugation (12,000 rpm for 30 min at 4°C), the concentration of the supernatant was normalized using the BCA protein assay kit. We examined the levels of vascular endothelial growth factor C (VEGF-C) and Aβ1–42 using commercial ELISA kits (Elabscience Biotechnology Co., Ltd) according to the manufacturer’s instructions. Absorbance readings at 450 nm were acquired via a microplate reader.

### RNA extraction and real-time quantitative polymerase chain reaction (RT-PCR)

Total RNA was extracted using an RNA extraction kit (#R0026, Beyotime). RNA integrity was verified by 260/280 nm absorbance ratios using NanoDrop 2000 (Thermo Fisher Scientific). cDNA synthesis was obtained using a reverse transcription kit (#AG11706, ACCURATE BIOTECHNOLOGY (HUNAN)CO., LTD), and RT-PCR was performed using SYBR Green Master Mix (#AG11719) according to the manufacturer's protocol. The relative mRNA expression levels were determined using the 2^∧^(-ΔΔCt) method. Primer sequences are detailed in supplemental Table S2.

### Immunofluorescence

Sections were incubated with blocking buffer for 1 h at room temperature, then incubated with primary antibodies overnight at 4°C. The sections were then incubated with corresponding Alexa Fluor-conjugated secondary antibodies at room temperature for 1 h and coverslipped with DAPI reagent (ab104139, Abcam). Quantification of positive cells was performed using the ImageJ software. Six slices per brain and six fields per slice were chosen randomly at 20× magnification to analyze the stained cells.

### Nissl staining

Sections were stained with 0.5% in cresyl violet (#C0117, Beyotime) for 2 h at 37°C. The sections were then dehydrated in a graded series of ethanol (70%, 80%, 90%, and 100%), cleared in xylene, and coverslipped using Permount. Neuronal density was estimated using stereological principles as described previously (23, 24). Quantification was performed every 8 sections. The optical dissector was obtained using a 40× objective, and the size of the counting frame was 120 × 120 μm. The estimated number of neurons in the section was obtained with a section thickness of 50 μm.

### Statistical analysis

The number of animals is represented in the legend as n = × mice per group. All data are expressed as the mean ± SEM. Statistical analysis was performed using GraphPad Prism v8.0.1. The detailed statistical methods are explained in the figure legends. *P* < 0.05 indicates a statistical difference.

## Results

### Long-term Omega-3 PUFAs supplementation ameliorates age-related motor and cognitive decline

**Fig. 1:**
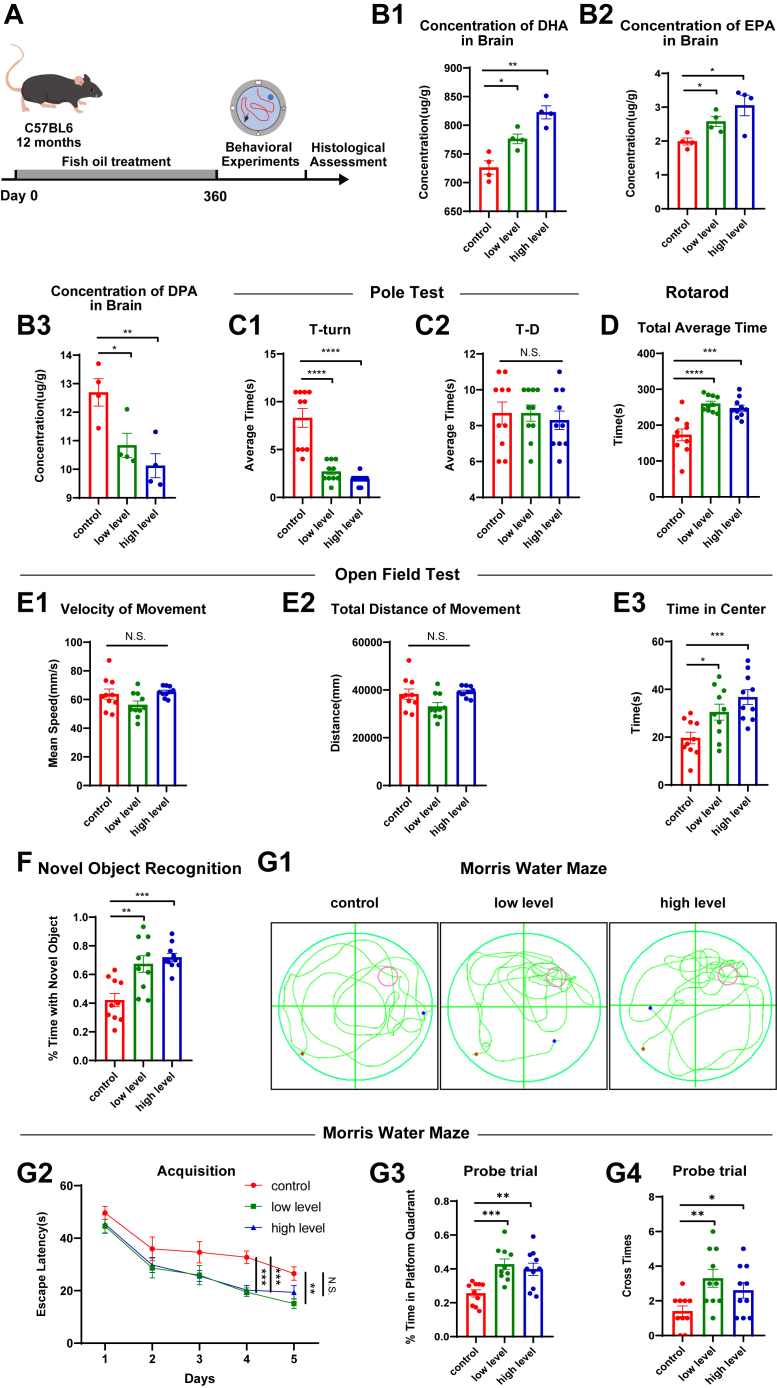
Long-term Omega-3 PUFA supplementation ameliorates age-related motor and cognitive decline. A: Schematic diagram of the experimental design. B: Major content of Omega-3 PUFAs quantified by GC-MS after a diet rich in Omega-3 PUFAs. (B1) DHA concentration (n = 4). (B2) EPA concentration (n = 4). (B3) DPA concentration (n = 4). C: Pole test results. (C1) Orientation latency (T-turn) (n = 10). (C2) Total descent duration (T–D) (n = 10). D: Rotarod endurance performance (latency to fall). (n = 10). E: Open-field test results. (E1) Locomotor velocity (n = 10). (E2) Ambulatory distance (n = 10). (E3) Center zone occupancy time (n = 10). F: Results of NOR experiment: Novel object exploration time/(total time) (n = 10). G: Morris water maze test results. (G1) Representative movement routes. (G2) Escape latency during the training period (n = 10). (G3) Target quadrant preference in probe trial (n = 10). (G4) Platform crossing frequency (n = 10). Data represent mean ± SEM. All data were subjected to normal distribution and homogeneity of variance tests. Data in G2 were analyzed using repeated measurement analysis of variance (ANOVA), while the remaining data were analyzed using one-way ANOVA. Tukey's post hoc test was used for further comparisons. N.S., no significant differences, ∗, *P* < 0.05, ∗∗, *P* < 0.01, ∗∗∗, *P* < 0.001, ∗∗∗∗, *P* < 0.0001.

**Supplementary Figure S1:**
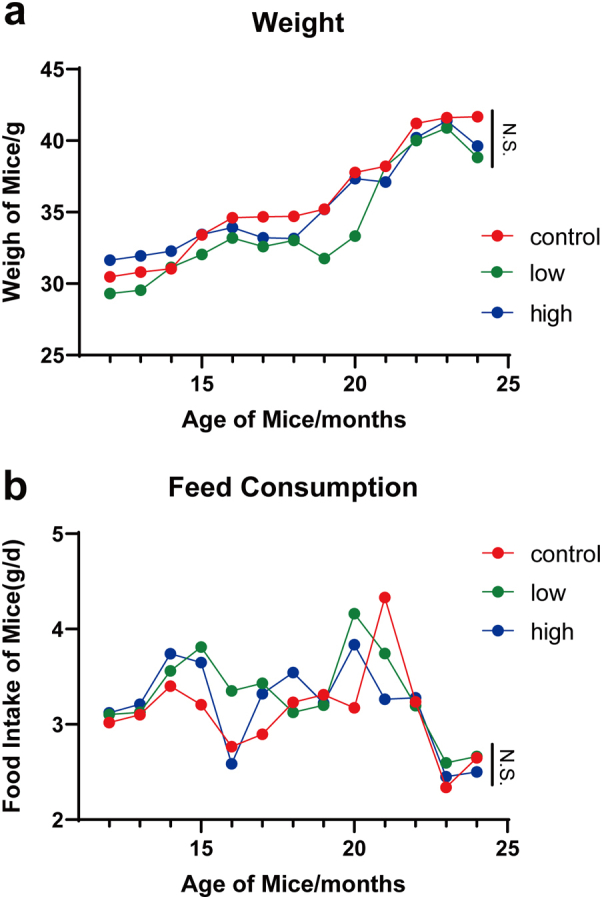


Prior research on the effects of dietary interventions in aging, particularly concerning omega-3 polyunsaturated fatty acids, has predominantly focused on short-term studies in younger animals (18). In this study, we conducted a 12-month dietary intervention in 12-month-old C57BL/6J mice using three distinct regimens: control (standard chow), low-dose (271.43 mg/kg fish oil), and high-dose (1,357.15 mg/kg fish oil) (supplemental Fig. S1). A series of behavioral assessments was systematically performed at study termination, followed by molecular and histopathological analyses (Fig. 1 a). GC-MS quantification confirmed dose-dependent brain enrichment of Omega-3 PUFAs, with the high-dose supplementation group showing substantially greater DHA and EPA accumulation compared to controls, while low-dose administration produced intermediate levels (Fig. 1 b1-b2). Consistent with these findings, DPA levels were reduced following omega-3 PUFA supplementation (Fig. 1 b3).

Pole test performance showed that mice from the two supplementation groups exhibited significantly reduced orientation latency (T-turn) versus controls (Fig. 1 c1-c2). The Rotarod experiment showed mice from the two supplementation groups maintained balance significantly longer than controls (Fig. 1 d), confirming improved motor coordination.

Open field testing revealed attenuated anxiety-like behavior in the two supplementation groups, as evidenced by increased central zone dwell time, with more pronounced effects observed in high-dose mice (Fig. 1 e3). In the NOR experiment, both supplementation groups displayed preferential exploration of the novel object, suggesting enhanced spatial and object memory abilities (Fig. 1 f). In the Morris water maze experiment, supplemented mice achieved faster platform localization on the fourth and fifth days of training (Fig. 1 g2). During the probe trial, increased target quadrant preference was observed in both supplementation groups, demonstrating improved learning and memory abilities of spatial position (Fig. 1 g3-g4).

### Long-term Omega-3 PUFAs supplementation reduces neuronal loss and decreases apoptosis in cortical and hippocampal tissues of aged mice

**Fig. 2:**
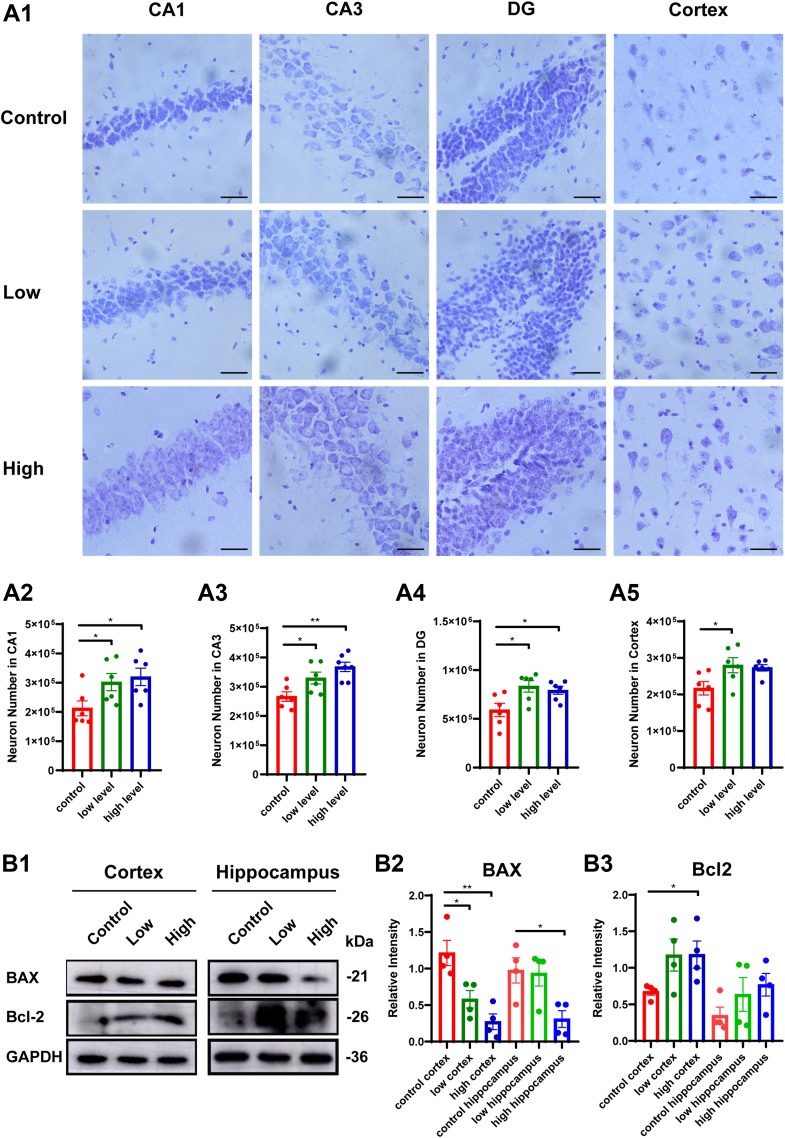
Long-term Omega-3 PUFA supplementation reduces neuronal loss and decreases apoptosis in aged mice. A: Stereological quantification of Nissl-positive neurons: (A1) Schematic representation of neurons in hippocampal subfields (CA1, CA3, DG) and cortical regions. (A2) CA1 neuronal density (n = 6). (A3) CA3 neuronal density (n = 6). (A4) DG neuronal density (n = 6). (A5) Cortical neuronal density (n = 6). B: Changes in the expression of apoptosis-related proteins in the brain parenchyma. (B1) Representative Western blot images of apoptosis-related proteins. (B2) Quantitative Bax expression (normalized to GAPDH) (n = 4). (B3) Quantitative Bcl-2 expression (normalized to GAPDH) (n = 4). Data represent mean ± SEM. All data were subjected to normal distribution and homogeneity of variance tests and then were analyzed using one-way ANOVA. Tukey's post hoc test was used for further comparisons. ∗, *P* < 0.05, ∗∗, *P* < 0.01. Scale: a1, 50 μm.

Stereological analysis on Nissl-stained sections revealed significant preservation of neuronal density in cortical and hippocampal regions of supplemented groups compared to age-matched controls (Fig. 2 a1-a5). This neuroprotective effect was particularly pronounced in CA1 and CA3 hippocampal subfields of high-dose animals, demonstrating Omega-3's capacity to attenuate age-related neuronal loss. Additionally, the quantification results of apoptosis-related markers showed decreased levels of the pro-apoptotic protein BAX and increased levels of the anti-apoptotic protein Bcl-2 in mice from both the supplementation groups (Fig. 2 b1-b3), indicating that Omega-3 PUFAs supplementation could mitigate apoptosis.

### Long-term Omega-3 PUFAs supplementation promotes cerebral clearance and meningeal lymphatic remodeling in aging

#### Enhanced clearance of metabolic products and pathoproteins

**Fig. 3:**
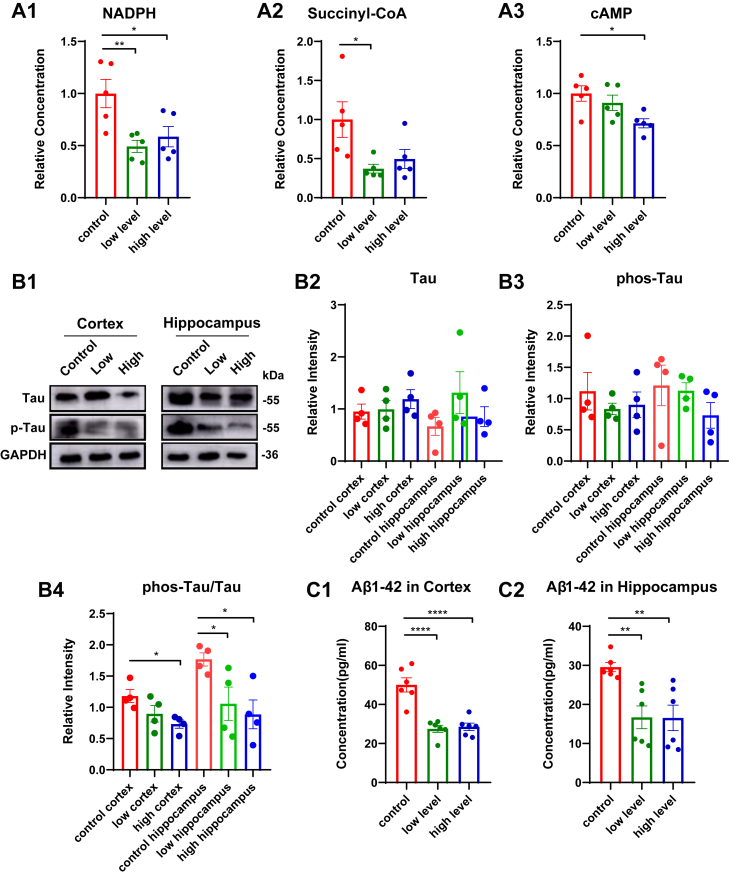
Long-term Omega-3 PUFA supplementation promotes cerebral clearance in aging. A: Content of metabolic products in the cerebrum homogenate of aged mice. (A1) NADPH content (n = 5). (A2) Succinyl-CoA content (n = 5). (A3) cAMP concentration (n = 5). B: Endogenous Tau protein levels in aged mice. (B1) Representative Western blot images of the total tau and phosphorylated tau in the cortex and hippocampus. (B2) Quantification of total tau protein (normalized to GAPGH; n = 4). (B3) Phosphorylated tau protein expression (normalized to GAPDH; n = 4). (B4) Ratio of phosphorylated tau protein to total tau protein (n = 4). C: Endogenous Aβ1-42 levels in aged mice. (C1) Cortical Aβ1-42 content (n = 6). (C2) Hippocampal Aβ1-42 content (n = 6). Data represent mean ± SEM. All data were subjected to normal distribution and homogeneity of variance tests and then were analyzed using one-way ANOVA. Tukey's post hoc test was used for further comparisons. ∗, *P* < 0.05, ∗∗, *P* < 0.01, ∗∗∗∗, *P* < 0.0001.

To elucidate the neuroprotective mechanisms of Omega-3 PUFAs, we systematically evaluated cerebral clearance through integrated biochemical and tracer-based assays. GC-MS profiling demonstrated enhanced clearance of metabolic products in the brains from both the supplementation groups, including NADPH (a redox regulator), succinyl-CoA (a tricarboxylic acid cycle intermediate), and cAMP (a critical secondary messenger) (Fig. 3 a1-a3), suggesting possible improved metabolites removal. Pathologically, NADPH accumulation might exacerbate oxidative neuronal damage via reactive oxygen species bursts (25), while retained succinyl-CoA disrupts mitochondrial energy homeostasis through metabolite inhibition (26). Dysregulated cAMP signaling could lead to abnormal cellular responses (27).

We further assessed clearance of toxic proteins. Tau protein analysis revealed stable total tau levels (Fig. 3 b2), while the ratio of p-Tau to total tau was significantly reduced in supplemented mice (Fig. 3 b2-b4), indicating selective clearance of pathological phosphorylated Tau protein. Complementary ELISA quantification showed decreased endogenous Aβ1-42 accumulation in both cortical and hippocampal regions of supplemented mice, consistent with enhanced proteopathic clearance (Fig. 3 c1-c2). Omega-3 significantly reduced cerebral accumulation of metabolites (NADPH, succinyl-CoA, cAMP) and pathoproteins (p-Tau, Aβ1-42). These improvements likely involve multi-system coordination.

#### Meningeal lymphatic structural remodeling as a potential mechanism

As primary conduits for CSF-ISF convective transport, meningeal lymphatics mediate CNS clearance of metabolic products and pathoproteins (4, 5). Building upon previous studies that have documented age-related regression of meningeal lymphatics, we next examined whether long-term supplementation with Omega-3 polyunsaturated fatty acids (PUFAs) could ameliorate these aging-associated changes.

**Fig. 4:**
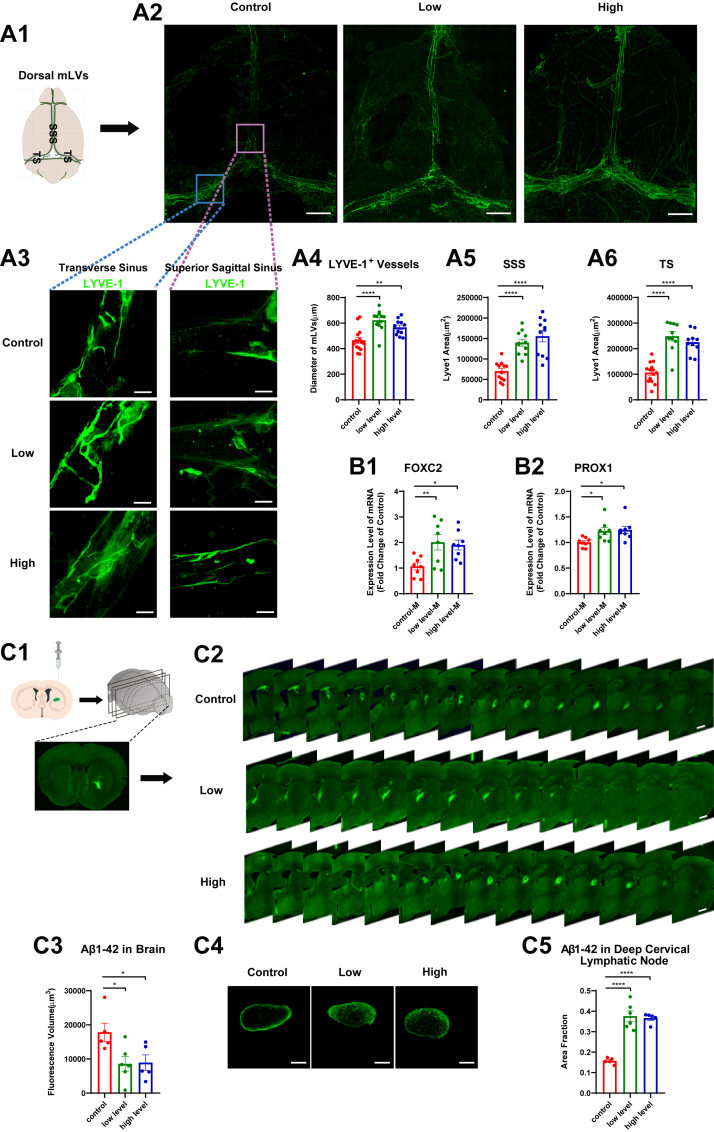
Long-term Omega-3 PUFA supplementation promotes meningeal lymphatic remodeling in aging. A: Morphometric analysis of MLVs. (A1) Schematic representation of MLVs. (A2) Whole-mount immunofluorescence of LYVE-1^+^ MLVs. (A3) High-magnification views of TS and SSS-associated MLVs. (A4) Quantification of MLV diameter (n = 10). (A5) MLV coverage area in SSS (n = 10). (A6) MLV coverage area in TS (n = 10). B: Expression of lymphatic markers in the meninges (M). (B1) Foxc2 mRNA expression (n = 8). (B2) Prox1 mRNA expression (n = 8). C: Clearance efficiency of exogenous Aβ1-42 in aged mice one day after injection. (C1) Stereotaxic injection schematic. (C2) Representation brain sections showing residual FITC-Aβ1-42. (C3) Quantified cerebral Aβ1-42 retention (n = 5–6). (C4) Representation of drained Aβ1-42 in dCLNs. (C5) Statistical results of fluorescence in dCLNs (n = 5–6). Data represent mean ± SEM. All data were subjected to normal distribution and homogeneity of variance tests and then were analyzed using one-way ANOVA. Tukey's post hoc test was used for further comparisons. ∗, *P* < 0.05, ∗∗, *P* < 0.01, ∗∗∗∗, *P* < 0.0001. Scale: a2, 1 mm; a3, 200 μm; c2, 3 mm; c4, 200 μm. Abbreviations: TS, Transverse sinus, SSS, Superior sagittal sinus.

**Supplementary Figure S2:**
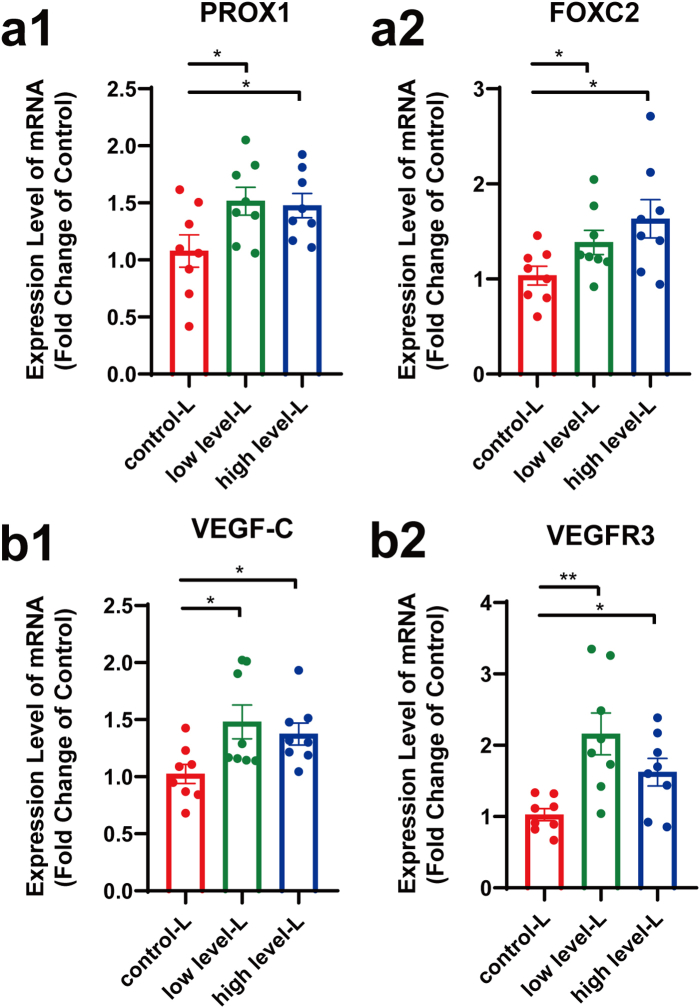


Morphological analyses revealed that long-term Omega-3 PUFA supplementation significantly attenuated age-associated regression of meningeal lymphatic vessels (MLVs). Morphometric analyses demonstrated substantial preservation of lymphatic architecture in supplemented groups, with improvements in both vessel diameter and network complexity compared to control animals (Fig. 4 a1-a6). Molecular characterization revealed coordinated upregulation of lymphatic maintenance programs. Transcriptional analysis identified marked elevation of *Prox1* expression in MLVs and dCLNs, a master regulator of lymphatic endothelial identity. Parallel increases in *Foxc2* levels within MLVs and dCLNs indicated enhanced valvular morphogenesis (Fig. 4 b1-b2, supplemental Fig. S2 a1-a2), suggesting the mitigation of atrophy in the MLVs.

#### Functional–structural correlation analysis

To directly assess meningeal lymphatic drainage, exogenous FITC-Aβ1-42 was stereotaxically injected into the left striatum of aged mice, mimicking extracellular Aβ deposition. Twenty-four hours post-injection, we collected brains and dCLNs and detected residual fluorescence of Aβ1-42. Supplemented groups exhibited significantly reduced cerebral Aβ retention with concomitant increased drainage to the dCLNs (Fig. 4 c1-c5), demonstrating enhanced meningeal lymphatic clearance efficiency and suggesting lymphatic remodeling may be one key mechanism underlying Omega-3 PUFAs' effects.

### Long-term Omega-3 PUFAs supplementation potentiates VEGF-C-driven meningeal lymphatic remodeling via PI3K-AKT-mTOR activation

**Fig. 5:**
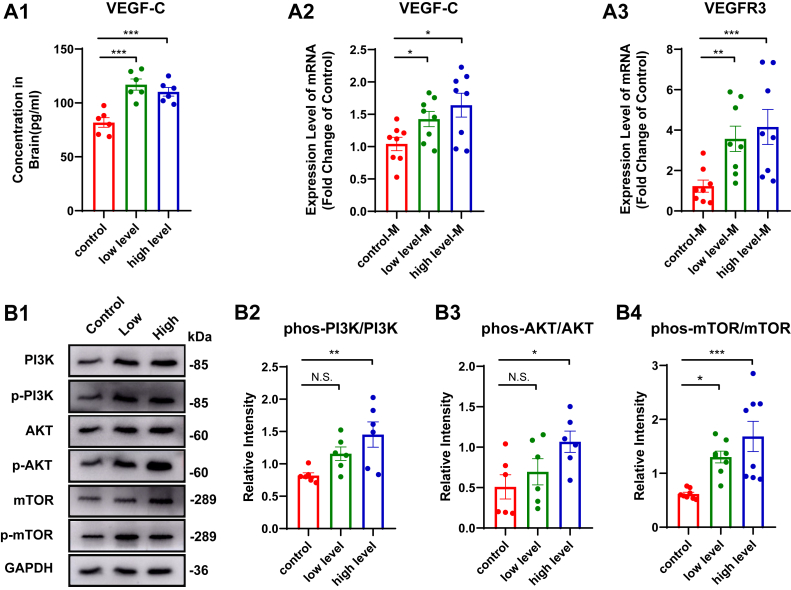
Long-term Omega-3 PUFA supplementation activates PI3K/AKT/mTOR/VEGF signaling to preserve meningeal lymphatic architecture. A: Expression of VEGF-C and its receptor VEGFR3. (A1) Whole-brain VEGF-C protein (n = 6). (A2) *Vegfc* mRNA expression levels in meninges (M) (n = 8). (A3) *Vegfr3* mRNA expression levels in meninges (M) (n = 8). B: The expression of proteins in the PI3K-AKT-mTOR pathway in the brains. (B1) Representative western-blot images of PI3K, phosphorylated PI3K, AKT, phosphorylated AKT, mTOR, and phosphorylated mTOR. (B2) p-PI3K/PI3K ratio (n = 6). (B3) p-AKT/AKT ratio (n = 6). (B4) p-mTOR/mTOR ratio (n = 6). Data represent mean ± SEM. All data were subjected to normal distribution and homogeneity of variance tests and then were analyzed using one-way ANOVA. Tukey's post hoc test was used for further comparisons. N.S., no significant differences, ∗, *P* < 0.05, ∗∗, *P* < 0.01, ∗∗∗, *P* < 0.001.

To investigate the mechanistic basis of Omega-3-mediated lymphatic preservation, we focused on VEGF-C/VEGFR3 signaling - a master regulatory pathway for meningeal lymphatic development (6, 28). Given that VEGF-C is secreted by smooth muscle cells (SMCs)—which are abundant in brain parenchyma—as well as by the pineal and pituitary glands (6, 29), we propose its presence in brain parenchyma and subsequent transport to the meninges to exert effects. To verify upregulated VEGF-C expression, we concurrently measured VEGF-C protein in brain tissue and *Vegfc* mRNA in meninges, complemented by findings from peripheral dCLNs. Immunoassay quantification revealed coordinated upregulation of VEGF-C protein in whole-brain lysates and dCLNs in supplemented groups, paralleled by increased *Vegfc* mRNA expression in meninges and dCLNs (Fig. 5 a1-a2, supplemental Fig. S2 b1). What`s more, meningeal lymphatics and dCLNs in Omega-3-supplemented groups exhibited upregulated *Vegfr3* mRNA expression—the direct effector of VEGF-C signaling—supporting enhanced meningeal lymphatic responsiveness to VEGF-C stimulation for trophic maintenance of lymphatic endothelium (Fig. 5 a3, supplemental Fig. S2 b2).

To elucidate how Omega-3 PUFAs modulate VEGF-C expression, we considered established mechanisms by which Omega-3s are known to activate the PI3K-AKT-mTOR pathway (30, 31, 32). Existing research has confirmed that activation of this pathway could promote the expression of VEGF, subsequently influencing angiogenesis (31, 33). Consequently, we hypothesized that Omega-3 PUFAs might activate the PI3K-AKT-mTOR pathway, promoting the expression of VEGF-C, thereby inducing the expansion of MLVs.

To confirm this hypothesis, we measured the total and phosphorylated PI3K, AKT, and mTOR proteins in brains. The results demonstrated significant phosphorylation of PI3K, AKT, and mTOR proteins in supplemented groups compared to controls (Fig. 5 b1-b4). This post-translational modification profile indicates pathway activation, consistent with prior reports linking PI3K/AKT/mTOR signaling to VEGF transcriptional regulation.

### Long-term Omega-3 PUFAs supplementation modulates neuroimmune homeostasis of aged mice

**Fig. 6:**
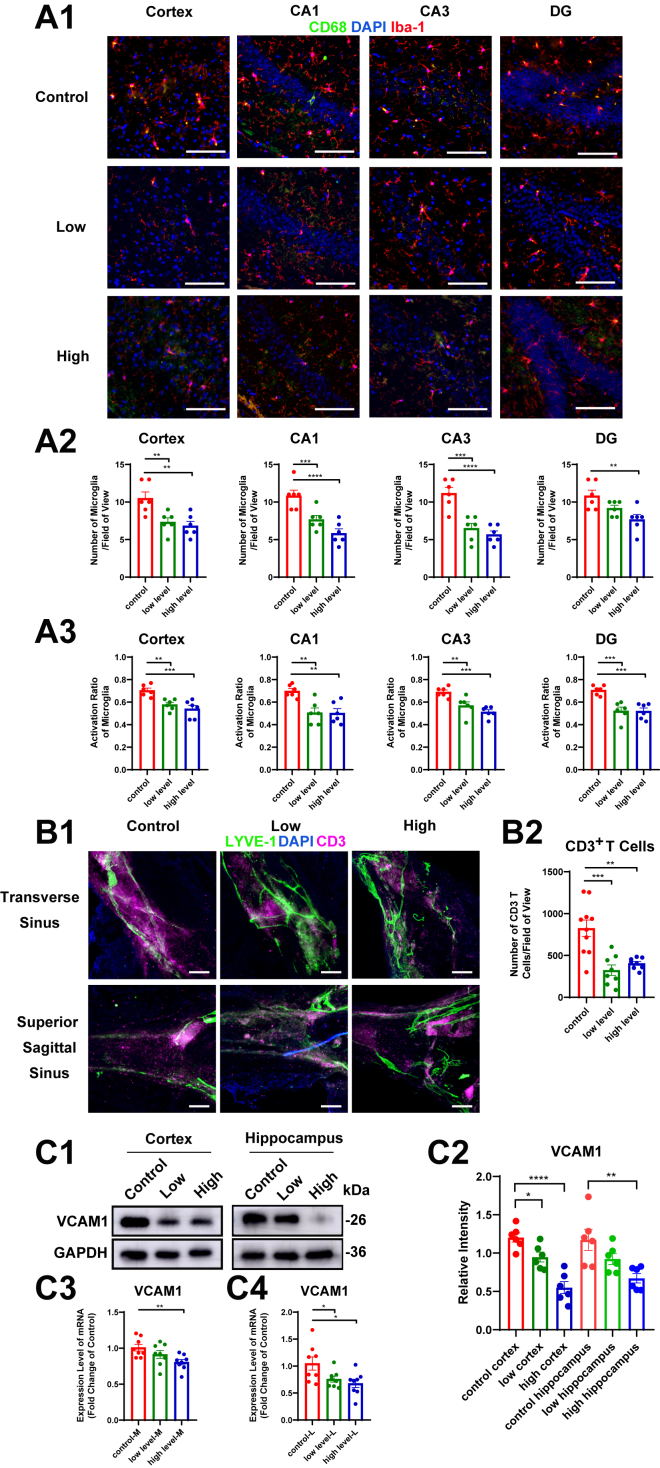
Long-term Omega-3 PUFA supplementation modulates neuroimmune homeostasis of aged mice. A: Total number and activation ratio of microglia in the cortex and hippocampus of aged mice. (A1) Representative cortical and hippocampal images. (A2) Quantification of parenchymal microglial density (n = 6). (A3) Activated microglia ratio (CD68^+^/Iba1^+^; n = 6). B: Meningeal T cell infiltration. (B1) Representative images of CD3+ T cells in the meninges. (B2) Quantified T cell density (n = 9). C: VCAM-1 expression profiling. (C1) Representative Western blot images of VCAM-1 protein in the cortex and hippocampus. (C2) VCAM-1 protein quantification (n = 6). (C3) Vcam1 mRNA expression levels in meninges (M) (n = 8). (C4) Vcam1 mRNA expression levels in dCLNs (L) (n = 8). Data represent mean ± SEM. All data were subjected to normal distribution and homogeneity of variance tests and then were analyzed using one-way ANOVA. Tukey's post hoc test was used for further comparisons. ∗, *P* < 0.05, ∗∗, *P* < 0.01, ∗∗∗, *P* < 0.001, ∗∗∗∗, *P* < 0.0001. Scale: a1, 200 μm; b1, 25 μm.

We subsequently investigated whether Omega-3 PUFAs supplementation exerted immunomodulatory effects during brain aging. Immunohistochemical analyses revealed a marked reduction in activated microglia populations across cortical and hippocampal regions in mice from both supplementation groups (Fig. 6 a1, a2), accompanied by decreased expression of lysosomal activation markers CD68 (Fig. 6 a1, a3). This phenotypic shift suggests microglia transition toward homeostatic surveillance states. Given MLVs’ newly identified role in immune cell transportation and age-related immune cell accumulation, we took T cells as peripheral immune indicators and found significant attenuation of CD3 expression within dural sinuses following Omega-3 PUFAs supplementation, indicative of reduced T cell accumulation (Fig. 6 b1, b2).

Then, we conducted preliminary experiments to study the underlying mechanisms. The increased level of VCAM-1 secreted by matrix cells associated with the aging meninges makes T cells prone to aggregate within the dura mater sinuses (13). Multiregional profiling showed consistent VCAM-1 downregulation in hippocampal, cortical, meningeal, and dCLN tissues in both the supplementation groups (Fig. 6 c1-c4), indicating that Omega-3 PUFAs supplementation influenced the expression of VCAM-1 on the meninges and brain. The suppression of VCAM-1 probably provides a molecular basis for observed reductions in meningeal T cell accumulation.

## Discussion

The neuroprotective potential of Omega-3 PUFAs in aging has been evidenced through preclinical models demonstrating reduced cortical atrophy and white matter preservation (17, 34, 35, 36, 37). However, clinical trials based on diverse populations yield conflicting results (9, 37, 38, 39), potentially attributable to variations in intervention timing and duration. Recognizing the progressive and irreversible nature of aging, early and long-term interventions may optimize therapeutic efficacy against age-related neurological decline (40). Therefore, our study implemented a preventive strategy through early-onset, sustained Omega-3 intervention in middle-aged mice—initiating supplementation at 12 months and maintaining it for 12 months - a paradigm designed to mirror preventive approaches in human aging trajectories (41).

Subsequently, we validated the anti-aging effects of Omega-3 PUFAs based on both functional and pathological changes. In agreement with prior research (17), our findings demonstrated that Omega-3 PUFAs supplementation increased the level of Omega-3 PUFAs in the cortex of both low-dose and high-dose supplementation groups. Moreover, long-term Omega-3 supplementation mitigated cognitive and motor deterioration while reducing neuronal loss and apoptosis, reaffirming that early application of Omega-3 PUFAs could contribute to decelerating the process of brain aging and enhancing overall brain function. Notably, the low-dose supplementation demonstrated comparable efficacy to high-dose supplementation across multiple experiments, suggesting a non-linear dose-response relationship. The findings imply that optimal neuroprotection may be achieved at moderate Omega-3 levels, potentially reducing risks of bleeding and lipid peroxidation associated with high-dose supplementation.

While Omega-3 PUFAs have demonstrated neuroprotective effects against brain aging, their precise mechanisms of action remain incompletely understood. Emerging evidence indicates that the meningeal lymphatic system plays a crucial role in clearing interstitial solutes and neurotoxic proteins from the central nervous system (4, 5, 42). This drainage function becomes progressively impaired during brain aging (8), and such lymphatic dysfunction has been implicated in the pathogenesis of various brain age-related diseases (28). Supporting this concept, experimental enhancement of meningeal lymphatic-mediated CSF clearance has been shown to ameliorate AD-like phenotypes in murine models (10). Our previous findings further demonstrated that elevated brain levels of Omega-3 PUFAs facilitated the clearance of exogenous soluble Aβ in CSF, consequently attenuating Aβ-induced neurotoxicity (18).

However, the functional relevance of meningeal lymphatics in brain aging and neurodegeneration remains under debate, as a study has reported minimal effects of lymphatic manipulation on disease progression (43), and the potential relationship between Omega-3 PUFAs and the meningeal lymphatic function in brain aging remains unexplored. To address the issues, our study aimed to investigate whether Omega-3 PUFA supplementation modulates meningeal lymphatic function and whether such modulation contributes to improved brain metabolites clearance in aging. Our findings indicate that Omega-3 supplementation significantly reduces cerebral accumulation of metabolic products, pathological tau phosphorylation, and endogenous Aβ peptides, while simultaneously enhancing exogenous Aβ clearance in aged mice. These functional improvements were accompanied by measurable morphological changes in the MLVs, including increased vessel diameter and expanded network coverage. Together, these findings suggest that Omega-3 PUFAs may attenuate age-related decline in meningeal lymphatic function, thereby preventing the pathological accumulation of neurotoxic substances and potentially slowing brain aging processes. However, we acknowledge that this study does not establish a direct causal relationship between improved meningeal lymphatic function and the observed neuroprotective effects, as no specific blockade or genetic manipulation of lymphatic pathways was performed. Future studies will be needed to determine whether these structural changes are necessary for the benefits conferred by Omega-3 supplementation.

Central to this lymphatic remodeling is VEGF-C-mediated signaling. VEGF-C, a recognized lymphangiogenic factor, is not only crucial for developmental lymphangiogenesis (28) but essential for meningeal lymphatic maintenance in adulthood, especially in diseases (44, 45). Our data demonstrate that Omega-3 PUFA supplementation upregulates VEGF-C expression, resulting in MLV expansion and consequent improvement in brain clearance capacity. Our subsequent literature review revealed that Omega-3 PUFAs are known to activate downstream signaling cascades, including the PI3K-AKT-mTOR pathway, which is a critical regulator of VEGF expression and lymphatic development (32, 46). This activation is often mediated through specific receptors for polyunsaturated long-chain fatty acids (47, 48), which have been proposed to play crucial roles in regulating metabolism and inflammation, thus influencing the process of brain-aging (47). Importantly, the results demonstrated that Omega-3 PUFA supplementation could specifically enhance phosphorylation of PI3K, AKT, and mTOR, culminating in elevated VEGF-C production within the CNS. While cortical PI3K/AKT/mTOR phosphorylation coincided with VEGF-C upregulation, we emphasize this correlation does not preclude other pathways, such as the ERK 1/2 pathway (49). Collectively, our data implicate cortical PI3K-AKT-mTOR as a likely driver of VEGF-C-mediated meningeal lymphatic remodeling; we acknowledge that the activity of the meningeal pathway has not been fully confirmed. Conditional knockout (e.g., SMC-specific PI3K KO) will be essential for causal validation, though current data provide a robust mechanistic hypothesis.

Additionally, emerging evidence highlights the meningeal lymphatic system as a critical mediator of CNS immune surveillance (13). Ligation of MLVs has been shown to promote T lymphocyte accumulation in the meninges (4, 5), a phenomenon that becomes more pronounced with advancing age (13). This age-related immune dysregulation is further compounded by microglial proliferation and activation (50), which also promotes T cell infiltration into the brain parenchyma by releasing chemotactic factors and upregulating the expression of VCAM-1 on endothelial cells, ultimately disrupting neuroimmune homeostasis (50, 51). Our results demonstrate that long-term Omega-3 PUFAs supplementation attenuates age-related immune dysregulation by simultaneously modulating both the brain parenchyma and meningeal lymphatic compartments. This includes the suppression of microglial activation, reduced accumulation of meningeal T cells, and downregulation of VCAM-1 expression. While the precise contribution of MLVs requires further investigation, our data support Omega-3 PUFAs` role in preserving neuroimmune homeostasis through multi-compartmental modulation. Our future investigations will be focused on elucidating the precise molecular mechanisms through which Omega-3 PUFAs regulate immune homeostasis in the aging brain.

## Conclusions

In summary, our findings establish that aging of the meningeal lymphatic system may contribute to cerebral senescence and age-related neurodegeneration. The data demonstrate that long-term Omega-3 PUFAs supplementation is associated with improved MLV function, as evidenced by enhanced toxic protein clearance and neuroimmune homeostasis preservation within the CNS. We have also explored the potential molecular mechanisms underlying the improvement of clearance, suggesting (but not proving) MLV modulation as a therapeutic axis. Future studies employing MLV-specific genetic models are needed to establish causal relationships. Taken together, these findings may offer new perspectives for the development of clinical strategies targeting early neurodegenerative changes.

## Ethics approval and consent to participate

Study procedures received approval from the ethics committees of the Sun Yat-Sen University (SYSU-IACUC-2022-000342, SYSU-IACUC-2021-000248).

## Data availability

The data will be made available upon request from the corresponding author.

## Supplemental data

This article contains supplemental data.

## Conflict of interest

The authors declare that they do not have any conflicts of interest with the content of this article.

## Figures and Tables

**Supplementary Figure S3:**