# Supplementary material for: Long-term Omega-3 polyunsaturated fatty acid supplementation improves meningeal lymphatic function during brain aging in mice
Source: J Lipid Res. 2025 Sep 9;66(11):100895. doi: 10.1016/j.jlr.2025.100895 (PMC12593584; doi:10.1016/j.jlr.2025.100895)
Supplement: Supplementary Figures and Tables [file mmc1.docx]

**Supplementary Material**

Title: Long-term Omega-3 Polyunsaturated Fatty Acid Supplementation Improves Meningeal Lymphatic Function During Brain Aging in Mice

Zhoujing Liu^a^, Jiamin Peng^a^, Xuemin Wang^a^, Fei Yin^b^, Fengjuan Su^c^, Zhong Pei^c*^, Hongfu Wu^d*^, and Chuanming Luo^a*^

Figure S1. Changes in weight and food consumption in control, low-dose, and high-dose Omega-3 PUFAs supplementation groups of aged mice.

Figure S2. Long-term Omega-3 PUFAs supplementation resulted in increased expression of lymphatic markers, VEGF-C, and VEGFR3 in deep cervical lymph nodes.

Figure S3. The full uncropped Gels and Blots images in the figures.

Table S1. Component of AIN-93G diet.

Table S2. A detailed summary of the primers used in RT-PCR.

Table S3. A list of abbreviations in article.


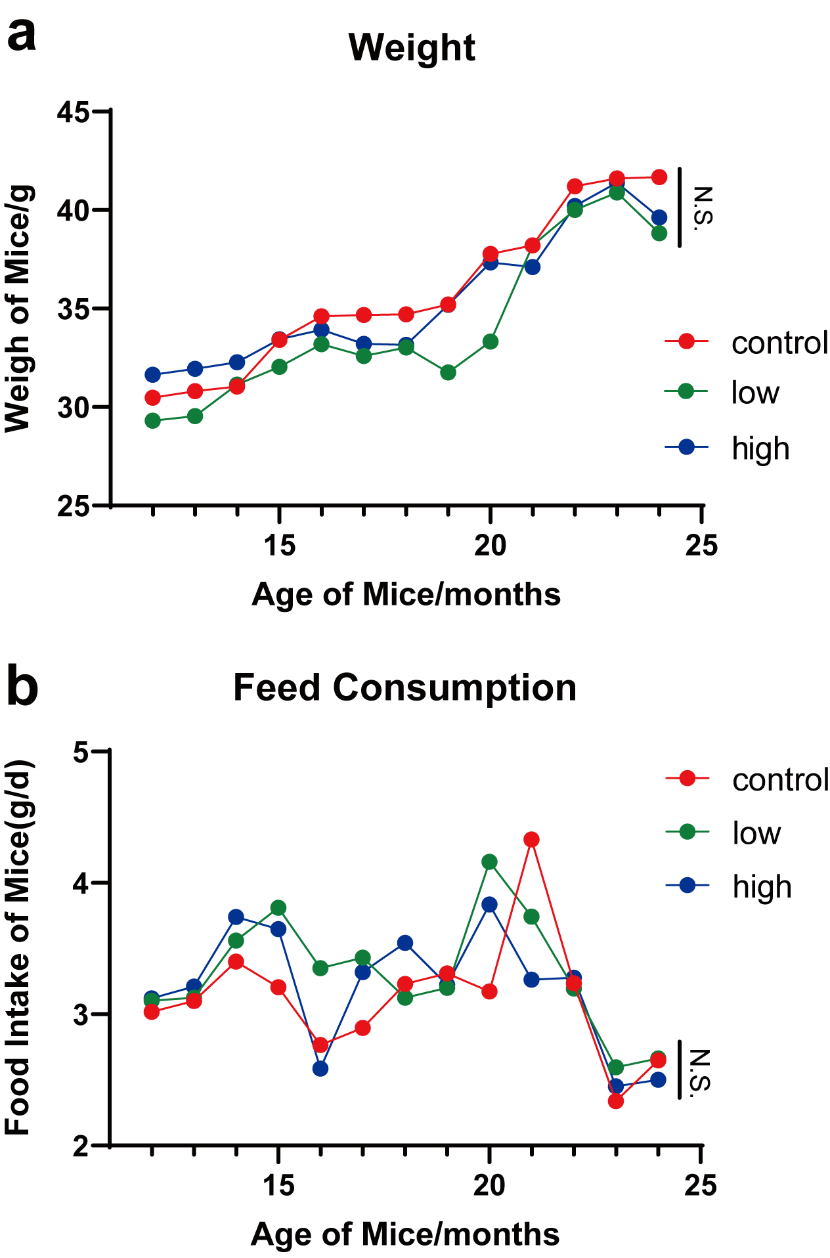


**Figure S1. Changes in weight and food consumption in the control, low-dose, and high-dose Omega-3 PUFAs supplementation groups of aged mice.**

(a) Trends in weight changes in aged mice during a diet rich in Omega-3 PUFAs.

(b) Trends in food consumption changes in aged mice during a diet rich in Omega-3 PUFAs.

All data were subjected to normal distribution and homogeneity of variance tests and were then analyzed using repeated measurement ANOVA. Tukey's post-hoc test was used for further comparisons. N.S., no significant difference.


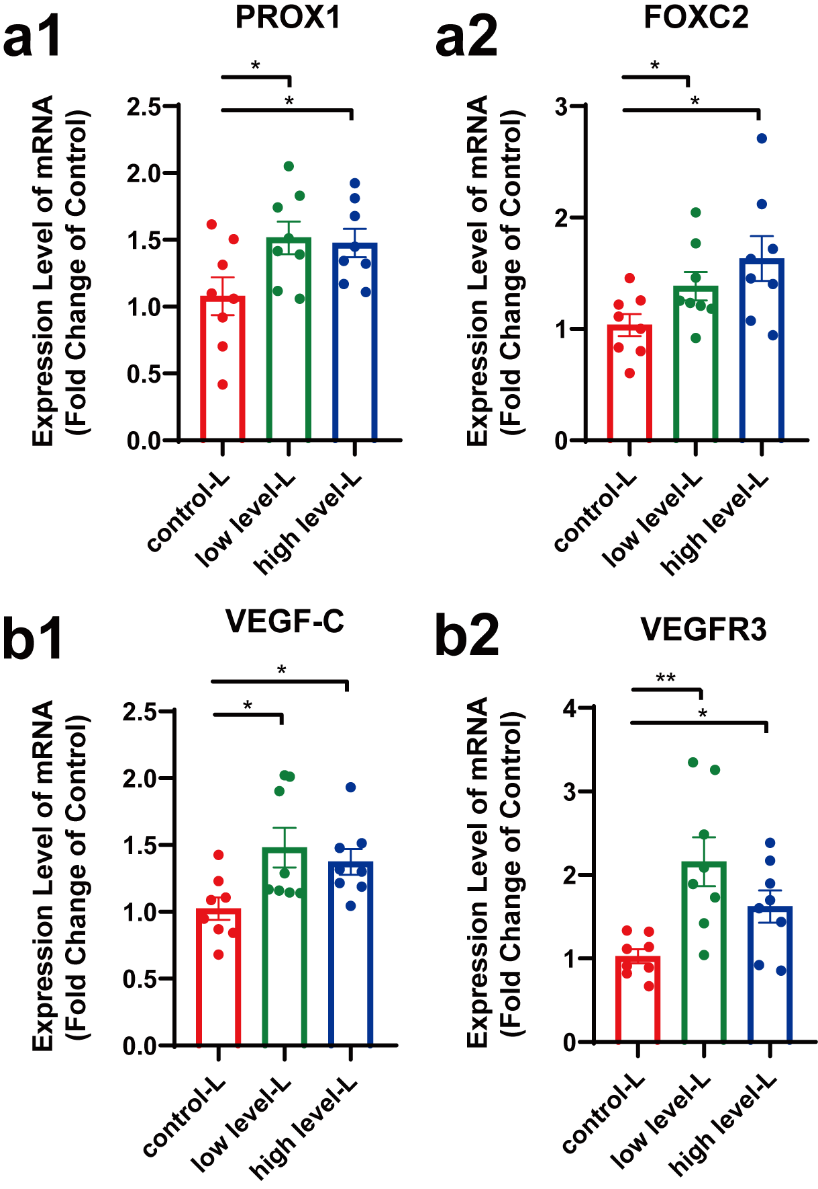


**Figure S2. Long-term Omega-3 PUFAs supplementation resulted in increased expression of lymphatic markers, VEGF-C, and VEGFR3 in deep cervical lymph nodes.**

(a) The expression of lymphatic markers in the deep cervical lymph nodes of aged mice from the control, low-dose, and high-dose of Omega-3 PUFAs supplementation groups. (a1) Statistical analysis of *Prox1* mRNA expression (n=8). (a2) Statistical results for *Foxc2* mRNA expression (n=8).

(b) Expression of VEGF-C and its receptor VEGFR3 in deep cervical lymph nodes. (b1) Statistical analysis of *Vegfc* mRNA expression in deep cervical lymph nodes (n=8). (b2) Statistical results of *Vegfr3* mRNA expression in deep cervical lymph nodes (n=8).

Data are means ± SEM. All data were subjected to normal distribution and homogeneity of variance tests and analyzed using one-way ANOVA. Tukey's post hoc test was used for further comparisons. *, *P*< 0.05, **, *P*< 0.01.


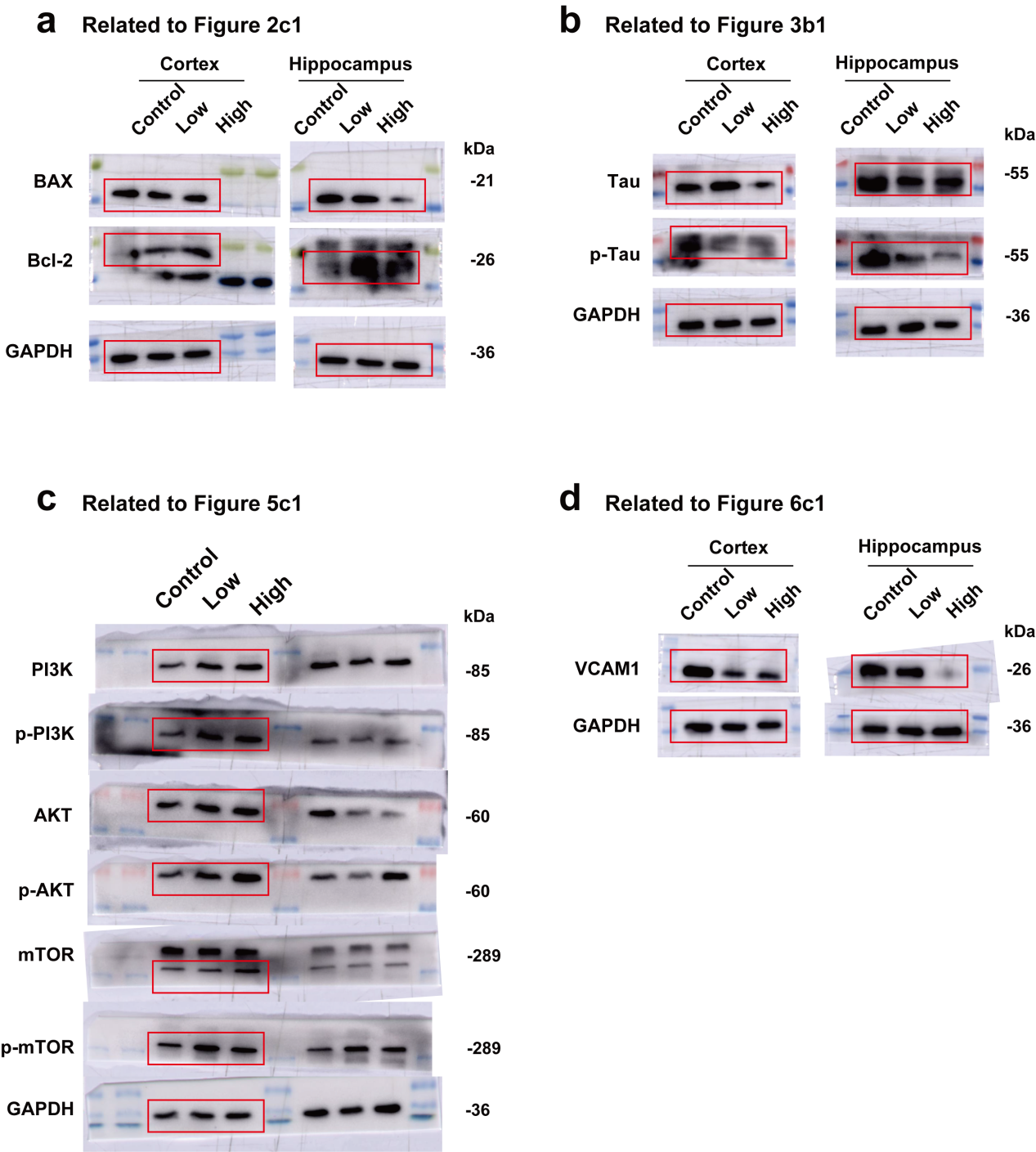


Figure S3. The full uncropped Gels and Blots images in the figures.

1. The full uncropped Blots images related to Figure 2c1.

(b) The full uncropped Blots images related to Figure 3b1.

(c) The full uncropped Blots images related to Figure 5c1.

(d) The full uncropped Blots images related to Figure 6c1.

**Table S1. Component of AIN-93G diet.**

| **Ingredient** | **Weight (g)** |
| --- | --- |
| Casein | 200 |
| L-Cystine | 3 |
| Corn-Starch | 397.5 |
| Maltodextrin | 132 |
| Sucrose | 100 |
| Cellulose | 50 |
| Soybean Oil | 70 |
| Vitamin Mix V10037 | 10 |
| Mineral Mix S10022G | 35 |
| Choline Bitartrate | 2.5 |
| Total | 1000 |

**Table S2. A detailed summary of the primers used in RT-PCR.**

| **Gene** | **Forward primer** | **Reverse primer** |
| --- | --- | --- |
| VEGF-C | CTTCTTGTCTCTGGCGTGTTC | CATCTACGCTGGACACAGACC |
| FOXC2 | AAGGAGGCCGAGAAGAAAGTC | GTCATGATGGTCTCCACGCT |
| VCAM-1 | AGTTGGGGATTCGGTTGTTCT | CCCCTCATTCCTTACCACCC |
| PROX1 | AGAAGGGTTGACATTGGAGTGA | TGCGTGTTGCACCACAGAATA |
| GAPDH | AGGTCGGTGTGAACGGATTTG | TGTAGACCATGTAGTTGAGGTCA |
| VEGFR3 | CTGGCAAATGGTTACTCCATGA | ACAACCCGTGTGTCTTCACTG |

**Table S3. A list of abbreviations in article.**

| **abbreviation** | **Full name** |
| --- | --- |
| Omega-3 PUFAs | Omega-3 polyunsaturated fatty acids |
| AD | Alzheimer’s disease |
| CSF | Cerebrospinal fluid |
| dCLNs | Deep cervical lymph nodes |
| Aβ | Beta amyloid protein |
| VCAM-1 | Vascular cell adhesion molecule-1 |
| DHA | Docosahexaenoic acid |
| EPA | Eicosapentaenoic acid |
| ALA | Alpha-linolenic acid |
| CNS | Central nervous system |
| GC-MS | Gas chromatography tandem mass spectrometry |
| UHPLC | Ultra-high-performance liquid chromatography |
| ESI | Electrospray ionization |
| FITC | Fluorescein isothiocyanate |
| NOR | Novel object recognition |
| PFA | Paraformaldehyde |
| PVDF | Polyvinylidene fluoride membranes |
| ELISA | Enzyme-linked immunosorbent assay |
| VEGF-C | Vascular endothelial growth factor C |
| RT-PCR | Real-time quantitative polymerase chain reaction |
| HE | Hematoxylin eosin |
| ANOVA | Analysis of variance |
| VEGFR-3 | Vascular endothelial growth factor receptor 3 |
| GPR120 | G-protein-coupled receptor 120 |
